# Styrenated Oil Synthesis with Cyclic Carbonate Functional Groups on Polystyrene Segment

**DOI:** 10.3390/polym13142343

**Published:** 2021-07-17

**Authors:** Eser Bingöl, Ahmet Tuncer Erciyes

**Affiliations:** Department of Chemical Engineering, Istanbul Technical University, 34469 Maslak, Istanbul, Turkey; eserbingol@itu.edu.tr

**Keywords:** triglyceride oil, styrene, styrene-AGC copolymer, cyclic carbonate, poly (hydroxy urethane) s, APTES

## Abstract

In this study, an oil-modified copolymer of 4-[(prop-2-en-1-yloxy)methyl]-1,3-dioxolan- 2-one (AGC) with styrene was synthesized, and the resulting copolymer (OBMI-St-AGC) was silane functionalized by inserting (3-aminopropyl) triethoxysilane (APTES) into the polymer backbone. OBMI-St-AGC was prepared by using an oil-based macroinitiator (OBMI) obtained by the esterification of linseed oil partial glycerides (PGs) with 4,4-azobis-4-cyanopentanoyl chloride (ACPC). In the characterization, FTIR, ^1^H NMR, TGA, and DSC analyses were applied. The silane-functionalized copolymer (OBMI-St-AGC-APTES) was crosslinked through the sol–gel process, and its crosslinked structure was determined.

## 1. Introduction

Synthetic polymers are used in a wide range of industrial products because of their controllable synthesis in view of the required properties. However, synthetic polymers have disadvantages from the standpoint of economic and environmental issues. The increase and fluctuations in petroleum prices, along with their non-biodegradable characteristics, forced the investigation of replacing petroleum-based chemicals with renewable sources [1,2,3]. Vegetable oils are the most important renewable raw material, and they are found in a large number of applications, such as in the manufacture of cosmetics, lubricants, adhesives, plastics, coating, and composite materials [4,5].

It is well known that triglyceride oils are subjected to modification according to the end use of the resulting product. In this context, in our laboratory, a number of studies were performed on the modification of triglyceride oils with vinyl and non-vinyl monomers for coating purposes. In the case of the vinyl monomer, macroinitiator and macromer methods were developed [6,7,8,9,10,11,12]. A macroinitiator (OBMI) was prepared by the reaction of partial glycerides (PGs) with 4,4-azobis-4-cyanopentanoyl chloride (ACPC) through the esterification reaction. In this way, a macroinitiator containing thermolabile azo groups was obtained. Upon heating this initiator in the presence of a styrene monomer, free radicals formed by the decomposition of azo groups combined with monomers and polymer chains were formed on the oil moiety [6,7,8,9]. In the present study, this strategy was applied by using styrene and 4-[(prop-2-en-1-yloxy)methyl]-1,3-dioxolan-2-one (AGC) monomers with the resulting poly(St-co-AGC) chains carrying oil moieties coming from the PGs. In the other modification route known as the macromer strategy, which was applied in our previous studies, a vinyl group was combined with the PGs. For this purpose, the PGswere first prepared and combined with acrylic acid or methacrylic acid. Then, it was polymerized with styrene. Since the vinyl groups on the oil moiety can undergo free radical polymerization with the vinyl monomers, chemical oil modification was achieved [10,11,12]. As for non-vinyl monomers, methylolated abietic acid [13], isocyanates [14], (3-aminopropyl) triethoxysilane (APTES) and titanium(IV) isopropoxide [15], benzoxazine [16,17], and caprolactone [18] were used in modifications.

In a continuation of these studies in the present work, the previously mentioned process of obtaining styrenated oil was taken one step further, and functionalized styrenated oil was obtained. In this way, as a contribution to the literature, a versatile novelty was imparted to the styrenated oil production concept applied in our previous studies. There is no doubt that the new intermediate product, i.e., functionalized styrenated oil, can be used to obtain a new product with desired properties by further modification through its functional sites. For the preparation of functionalized styrenated oil, as stated above, we used AGC monomer together with styrene. Consequently, the cyclic carbonate-functionalized polymer, OBMI-St-AGC, was obtained. In order to show functional polymer characteristics, OBMI-St-AGC was further modified with APTES. In the literature, the functionalized cyclic carbonate polymers were reacted with amine functional compounds [19,20]. The ring-opening reaction of cyclic carbonates with amines resulted in the formation of hydroxyl–urethane linkages. Linear or crosslinked structures were also obtained, depending on the functionality of the amine compound [21,22].

As previously mentioned, in the present work, AGC copolymer with styrene was synthesized in the presence of a macroinitiator obtained through the reaction of PGs with ACPC. In this way, a bulky oil moiety was inserted into the St-AGC copolymer backbone. Thus, the resulting product can be considered an oil-modified poly(St-co-AGC) (OBMI-St-AGC). As an orientation study, for a coating material, the drying property of this oil-modified product was examined. Additionally, in order to obtain the silane-functionalized polymer (OBMI-St-AGC-APTES), OBMI-St-AGC was subjected to further reaction with APTES as an application of its functional polymer characteristics.

## 2. Materials and Methods

### 2.1. Materials

Refined linseed oil was purchased commercially (iodine value: 170). Styrene (St, 99%, Aldrich) was passed through a basic alumina column to remove the inhibitor. Diethyl carbonate (≥99%, Aldrich, Saint Louis, MO, USA), K_2_CO_3_ (≥99%, Merck, Saint Louis, MO, USA), (3-aminopropyl) triethoxysilane (≥99%, Aldrich), and 3(allyloxy)-1,2 -propanediol (≥99%, Aldrich) were used as received. The 4,4-azobis(4-cyanopentanoic acid) (ACPA ≥ 98.0%, Aldrich) product was converted to the acid chloride derivative 4,4′azobis(4-cyanopentanoyl chloride) (ACPC) by using thionyl chloride (≥99.0%, Sigma-Aldrich, Saint Louis, MO, USA) [23]. All of the other chemicals were of analytical grade and used as received.

### 2.2. Characterization

The Fourier transform infrared spectra (FT-IR) were recorded on a Bruker Alpha-P spectrometer. Proton nuclear magnetic resonance (^1^H NMR) spectra were recorded on an Agilent NMR System VNMRS 500 spectrometer at room temperature in CDCl_3_. Thermal gravimetric analysis (TGA) was performed on a Perkin Elmer Diamond TG/DTA with a heating rate of 10 °C/min under nitrogen flow (200 mL/min). Differential scanning calorimetry (DSC) was performed using a TA DSC Q2000 instrument with a heating rate of 10 °C/min under nitrogen flow (50 mL/min). The molecular weight of the copolymer was determined by using Agilent 1100 series gel permeation chromatography (GPC) with a refractive index (RI) detector. Polystyrene standards were used for calibration with the effective molecular weight range of 1000–500,000 g/mol. THF (with using butylated hydroxytoluene as a flow marker) was used as the eluent at a flow rate of 0.5 mL/min at 25 °C.

### 2.3. Preparation of Partial Glycerides (PGs)

Linseed oil and glycerol were used for the preparation of the PGs. Linseed oil and glycerol were taken into the reaction flask with a weight ratio of 1:0.085 and heated. Then, Ca(OH)_2_, which is 0.1% by wt of the oil portion (as a catalyst), was added at 218 °C. The temperature was increased to 230 °C, and the reaction was continued under the nitrogen atmosphere. The samples were taken from the reaction mixture periodically and poured into a three-fold amount of ethanol. When the alcohol solution became clear, the transesterification was terminated. After the reaction mixture was cooled, the mixture was dissolved in diethyl ether in the flask. It was first washed with 0.1% aqueous sulfuric acid solution and then with water to remove the catalyst and free glycerol. The ethereal solution was dried over anhydrous sodium sulphate, and the solvent was removed [24]. The hydroxyl and acid values of the PGs were determined [25]. The hydroxyl and acid values of the resulting PGs were found to be 130 mg KOH/g and 2.31 mg KOH/g, respectively.

### 2.4. Synthesis of Oil-Based Macroinitiator (OBMI)

An oil-based macroinitiator was synthesized according to our previous study [9]. An ACPC solution in dichloromethane was added dropwise to the PGs in pyridine at 0 °C. Then, the temperature was raised to 35 °C, and the reaction was continued for 80 h. The reaction mixture was then dissolved in diethyl ether. It was first washed with the 0.1% aqueous sulfuric acid solution, then with water, and, finally, dried with sodium sulphate. After the solvent was evaporated, OBMI was obtained and characterized by FT-IR and ^1^H NMR analyses.

### 2.5. Synthesis of 4-[(Prop-2-en-1-yloxy)methyl]-1,3-dioxolan-2-one (AGC)

The AGC was prepared using the method described by Zhu et al. [26]. Diethyl carbonate and 3-(allyloxy)-1,2-propanediol were added to the reaction flask with a weight ratio of 1:0.375. K_2_CO_3_ was added in the amount of 3.82% of the total mixture, and the system was heated to 120 °C. Then, the reaction was continued for 24 h while ethanol was distilled out by means of the Dean–Stark trap. Finally, the reaction mixture was distilled under reduced pressure, and the AGC was obtained and characterized by FTIR.

### 2.6. Synthesis of OBMI-St-AGC

The OBMI was added to the reaction flask containing xylene under an inert atmosphere. Styrene and AGC were added to the reaction flask 6 times each by wt of the OBMI and heated. The reaction mixture was stirred at 130 °C for 48 h. After this period, the reaction mixture was cooled, and the polymer was precipitated in methanol and filtered. Afterward, it was washed with methanol several times. The polymer was dried under a vacuum at 35 °C for 24 h. The obtained OBMI-St-AGC sample was characterized by FTIR and ^1^H NMR analyses.

### 2.7. Preparation of OBMI-St-AGC-APTES

The OBMI-St-AGC was dissolved in xylene and stirred for 20 min at room temperature. Under an inert atmosphere, the APTES was added to the flask in the amount of 5% wt of the OBMI-St-AGC, and the system was heated to 60 °C. The reaction mixture was stirred for 5 h at this temperature and then cooled. The polymer was precipitated in methanol, filtered, and then washed with methanol several times. The obtained polymer (OBMI-St-AGC-APTES) was dried under a vacuum at 35 °C for 24 h.

The OBMI-St-AGC-APTES was also crosslinked by means of the sol–gel process according to the procedures given in the literature [27,28]. The OBMI-St-AGC-APTES was dissolved in a mixture of xylene and methanol with a 1:1 weight ratio. The 1M HCl solution was added to this mixture in equal weight to the OBMI-St-AGC-APTES. The reaction mixture was stirred for 6 h at 50 °C and then cooled. The reaction mixture was poured into methanol. The undissolved portion was separated by filtration. The crosslinked OBMI-St-AGC-APTES (C-OBMI-St-AGC-APTES) polymer fraction was then dried under a vacuum at 45 °C for 24 h.

## 3. Results and Discussion

**Scheme 1:**
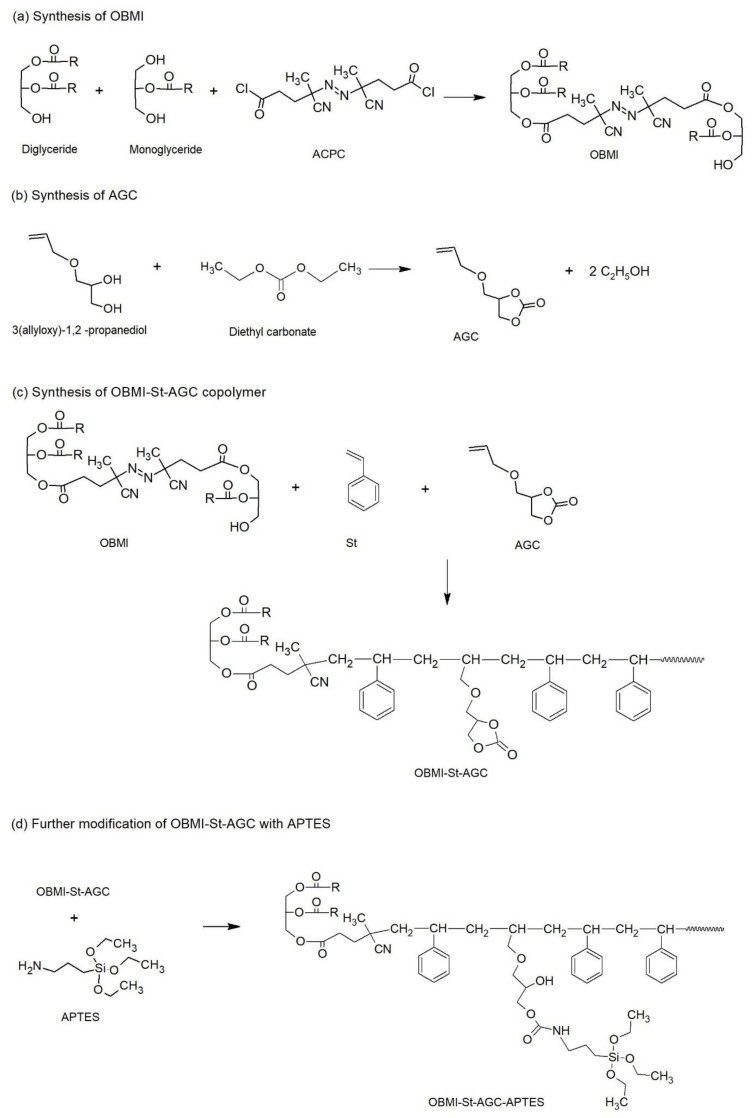
The synthesis of OBMI-St-AGC-APTES and its representative structure.

In this study, a novel copolymer with a five-membered cyclic carbonate structure was synthesized with free radical polymerization. The overall process is summarized in Scheme 1. First, the OBMI was synthesized by the esterification of PGs and ACPC (Scheme 1a), and then OBMI-St-AGC was synthesized via the radical polymerization of styrene and AGC in the presence of OBMI as an initiator (Scheme 1c). OBMI-St-AGC was further modified with APTES through the reaction of its NH_2_ with the cyclic carbonate group. In this way, OBMI-St-AGC-APTES was obtained as a final product (Scheme 1d).

Partial glycerides used in the synthesis of OBMI were prepared by the transesterification of linseed oil with glycerol. In this study, refined linseed oil was used. It contains approximately 9–10% palmitic acid, 7–8% stearic acid, 10–21% oleic acid, 13–15% linoleic acid, and 50–61% linolenic acid [4].

In order to eliminate the break formation during the transesterification reaction, refined grade linseed oil was used. Since the transesterification temperature is high enough, where even the oil contained a small amount of stabilized break, it is able to precipitate when reacted with glycerol during the transesterification reaction. This well-known feature of linseed oil is determined by the ISO 150 standard method [29]. It should be noted that in the present study, no precipitation was observed during the transesterification.

Furthermore, vegetable oils are subjected to the refining processes for both addible and technical purposes. In the refining process, minor components, such as free fatty acids, metals, chlorophylls, tocopherols and sterols, are removed [30,31,32,33,34].

Freedman et al. investigated variables affecting the yield of fatty esters from transesterified vegetable oils [30]. Their results showed that a complete conversion could be achieved in the alcoholysis reaction of refined cottonseed, peanut, soybean, and sunflower oils with methanol, ethanol, and butanol, whereas with crude oils, conversions were reduced due to the gums and extraneous materials present in the crude oils. These results showed that after the refining process, the minor components practically lost their effect on the transesterification reaction.

Therefore, in the present study, refined grade linseed oil was used as in the literature for the preparation of alkyd resin and urethane alkyd, where the transesterification reaction was applied [24,35,36].

In the transesterification reaction, PGs, namely, mono- and di-glyceride, are obtained. This reaction is carried out at an elevated temperature, and fatty acid radicals of triglycerides are randomly redistributed between the hydroxyl groups of glycerol and the newly-formed PGs. This is a reversible reaction, and the final product is a mixture of mono-, di- and tri-glycerides, where the percentages of which correspond to the final equilibrium state [37,38,39,40,41].

In the present study, under the applied transesterification conditions, the PGs were obtained with the hydroxyl and acid values of 130 mg KOH/g and 2.31 mg KOH/g, respectively. The composition of the PGs were determined to be 19% monoglyceride, 77% diglyceride, and 4% triglyceride [25]. The calculated molecular weight of the PGs were 576 with functionality of 1.33. According to this composition, OBMI (M_w_ = 1310 g mol^−1^ by GPC [9]) was not a linear structure and was formed by combining the acid ends of ACPC with one mole of PGs.

**Figure 1:**
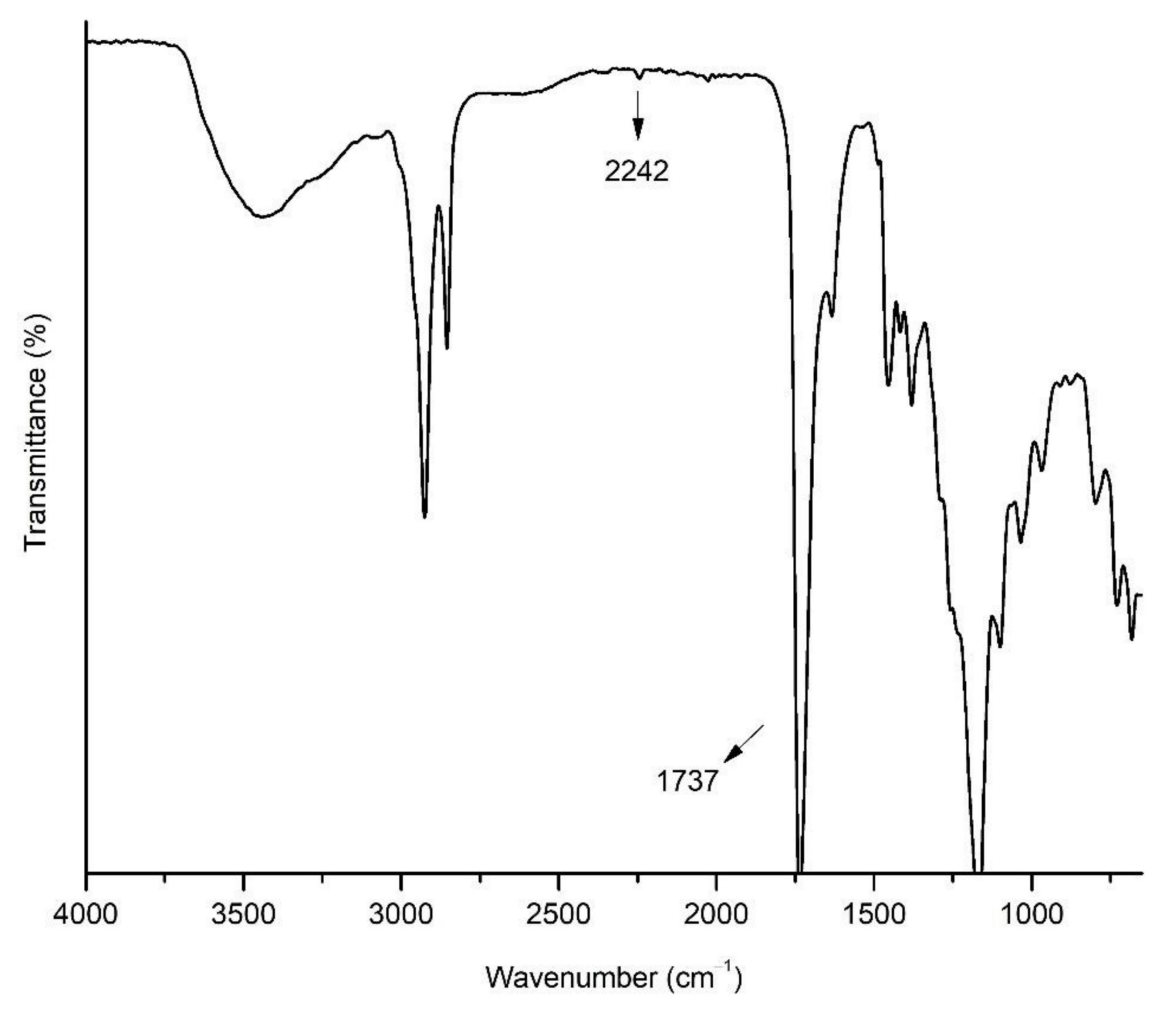
The FTIR spectrum of OBMI. (Perkin Elmer FTIR Spectrum One B Spectrometer).

**Figure 2:**
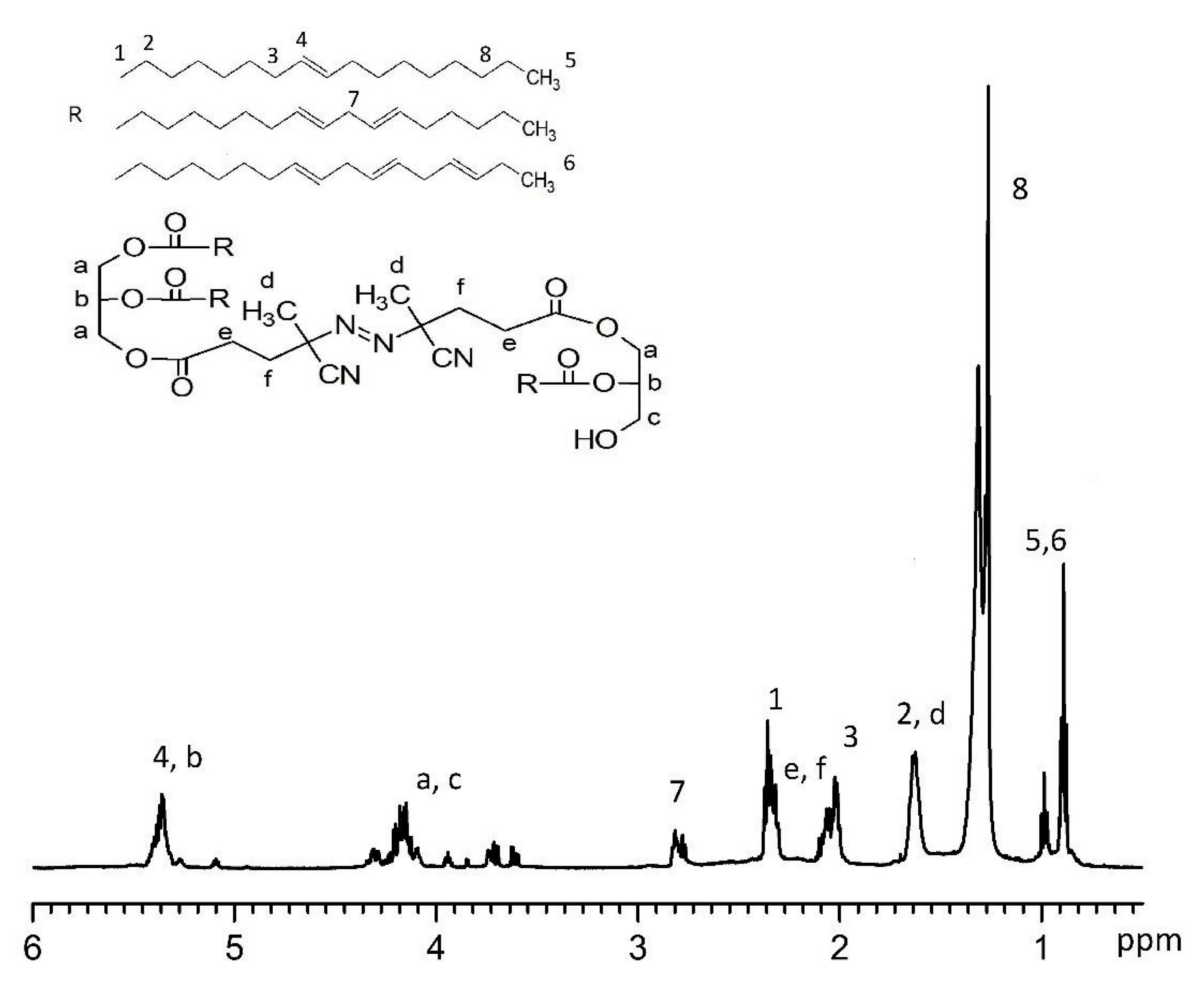
The ^1^H NMR spectrum of OBMI.

The FTIR spectrum of OBMI is given in Figure 1. The peak at 2242 cm^−1^ belongs to the CN group of ACPC. The ester peak at 1737 cm^−1^ shows the presence of oil moiety due to the PGs that are present in OBMI structure. These results showed that OBMI was successfully synthesized. The structure of OBMI was also confirmed by ^1^H NMR. The ^1^H NMR spectrum of OBMI is shown in Figure 2. The peaks at 3.5–4.3 and 5.4 ppm are attributed to the protons of the CH_2_-O-CO-R, CH_2_-OH, and CH-O-CO-R groups of oil moiety [9,42,43]. The peaks observed at 1–2.8 ppm resulted from the -CH_2_ and -CH_3_ groups present in OBMI [44].

**Figure 3:**
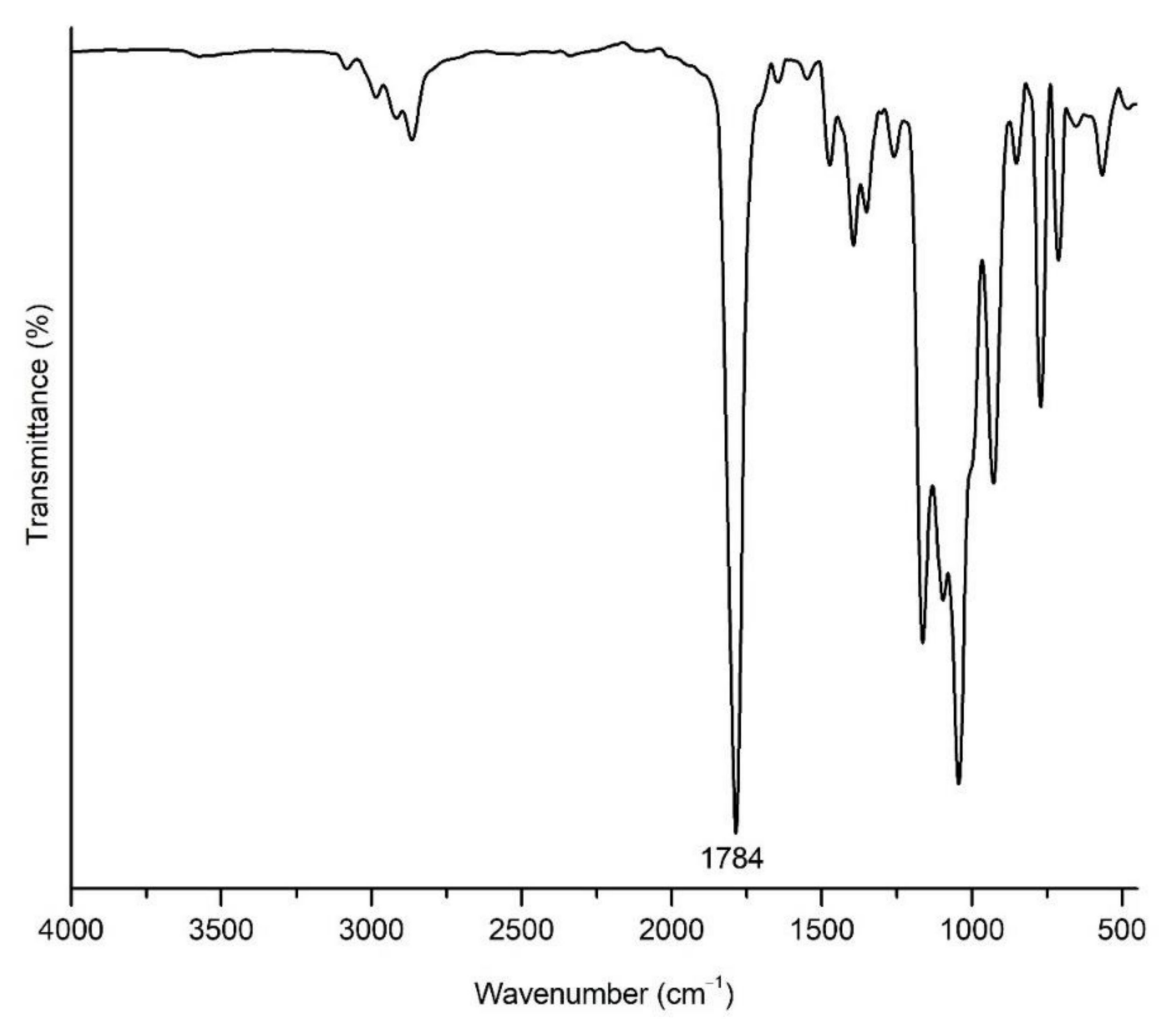
The FTIR spectrum of AGC.

The AGC, which was used in the OBMI-St-AGC preparation (Scheme 1b), was synthesized in the laboratory as explained in the literature [26]. The FTIR spectrum of the obtained AGC sample is given in Figure 3. The peak at 1784 cm^−1^ belongs to the C=O bond of the cyclic carbonate group, and because the C=O bond of the diethyl carbonate peak appears at 1740 cm^−1^, it was concluded that the synthesis was successfully achieved by evaluating and comparing this spectrum with the literature [45].

**Figure 4:**
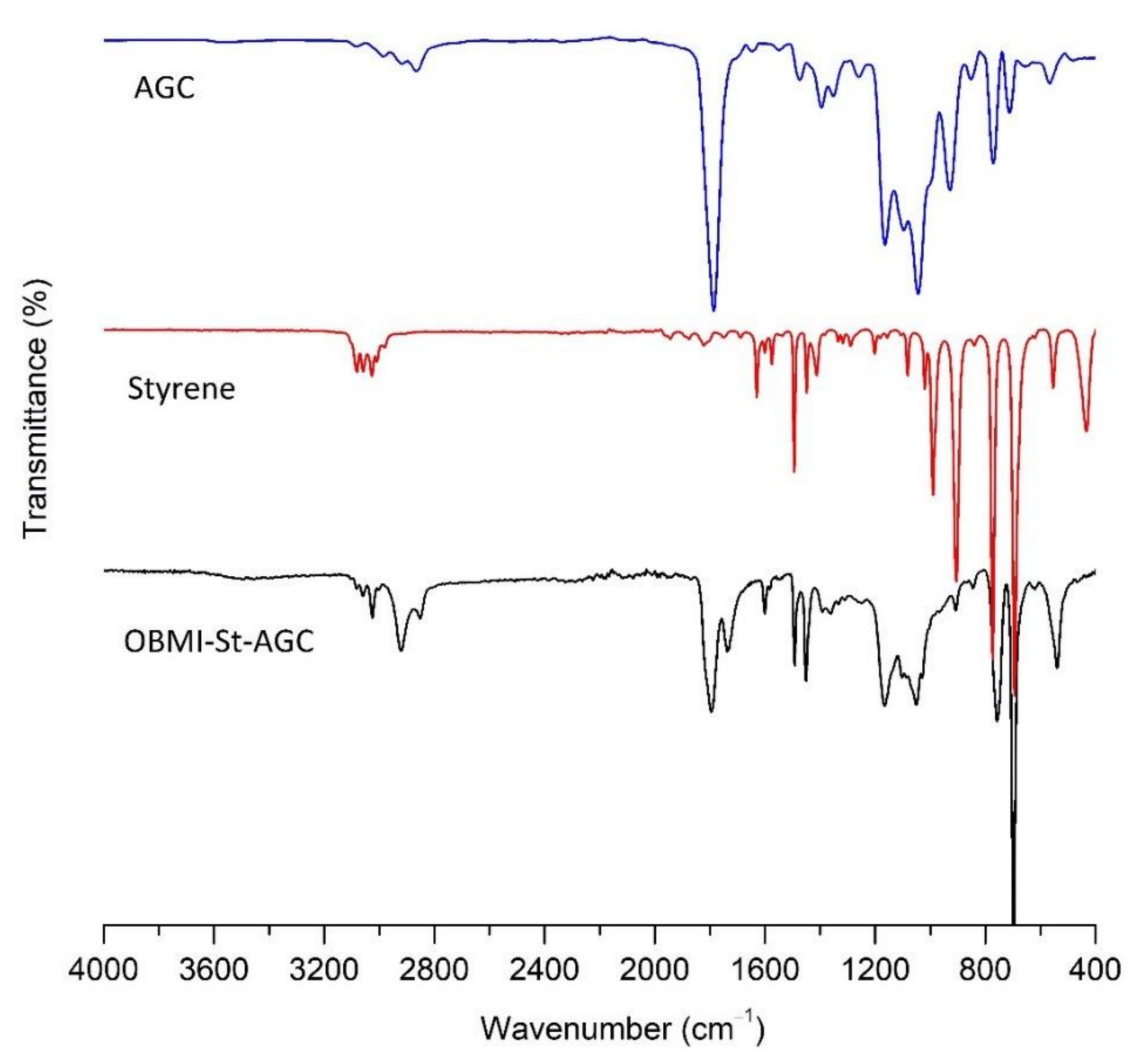
The FTIR spectra of AGC, styrene, and OBMI-St-AGC.

The representative structures of OBMI-St-AGC and OBMI-St-AGC-APTES are presented in Scheme 1c,d, respectively. The OBMI-St-AGC and OBMI-St-AGC-APTES samples are characterized by FTIR and ^1^H NMR analyses. These polymer samples were practically monomer free since they were purified by precipitation in methanol, as explained in Section 2.6 and Section 2.7. The FTIR spectrum of OBMI-St-AGC, AGC, and styrene are given in Figure 4, where the bands specific to styrene appeared at 1601 cm^−1^ (aromatic skeleton stretching of styrene),757 and 697 cm^−1^ (the pattern of five adjacent hydrogen atoms on the aromatic ring) [46,47]. The peak at 1795 cm^−1^ belongs to the C=O bond of the cyclic carbonate of AGC [48]. The C=O bond of oil moiety appears at 1737 cm^−1^ [49]. In comparison of the OBMI-St-AGC spectrum with the monomers spectra, the vinyl peak of styrene monomer at 1630 cm^−1^ [48] and the peaks at 997 and 928 cm^−1^ for allyl group of AGC [50] disappeared in the OBMI-St-AGC spectrum. These results showed that the free monomers were removed in the precipitation process, and the OBMI-St-AGC was successfully synthesized.

**Figure 5:**
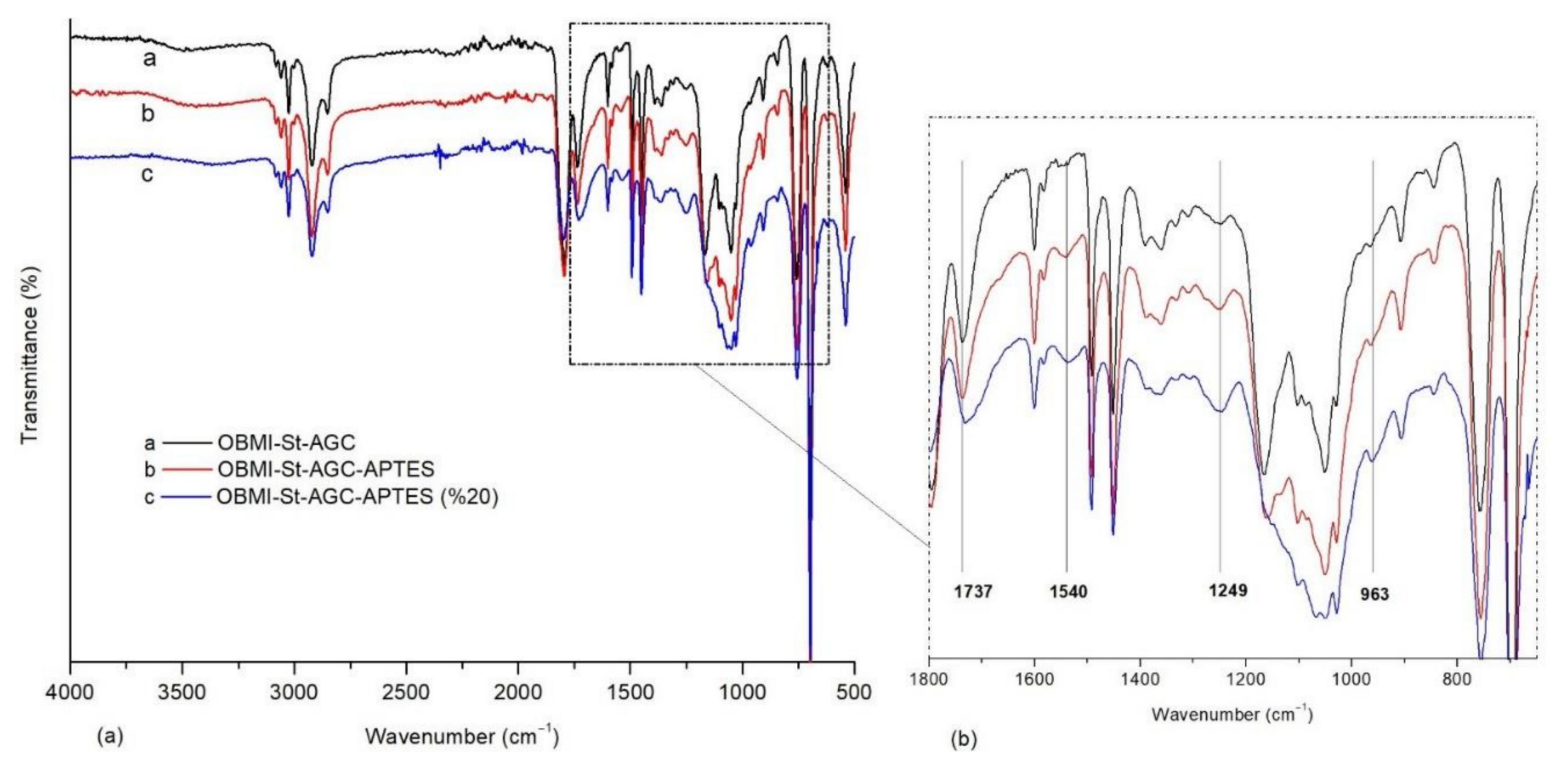
FTIR spectra of polymer samples (**a**); the enlarged peaks of the 1800–650 cm^−1^ region (**b**).

In order to understand whether the APTES could be incorporated into the OBMI-St-AGC structure, which shows the functional characteristics of OBMI-St-AGC, the OBMI-St-AGC-APTES samples were prepared with increased APTES amounts to make the APTES peaks discernible in the FTIR spectrum. In Figure 5, the FTIR spectra of the samples prepared by using % 5 and % 20 APTES are given together with that of OBMI-St-AGC. The intensity of the peak at 1540 cm^−1^ belonging to NH of urethane groups increased with the increasing APTES amount. The peak at 1737 cm^−1^, which belongs to C=O groups of oil moiety of OBMI-St-AGC, broadened due to the formation of a urethane structure [49,51,52]. The peak at 1249 cm^−1^ became larger as the amount of APTES increased, showing the formation of the urethane group [49].

Additionally, in the comparison of spectrum a, b, and c in Figure 5, the change in the peaks’ intensity at the 1000–1200 cm^−1^ region is due to the incorporation of Si-O-C and CH_2_-Si by means of APTES, and the increase in Si-O peak intensity at 963 cm^−1^ is due to APTES incorporation [15,53]. These results showed that the APTES was successfully combined with the OBMI-St-AGC chain through the reaction of its NH_2_ with cyclic carbonate groups.

**Figure 6:**
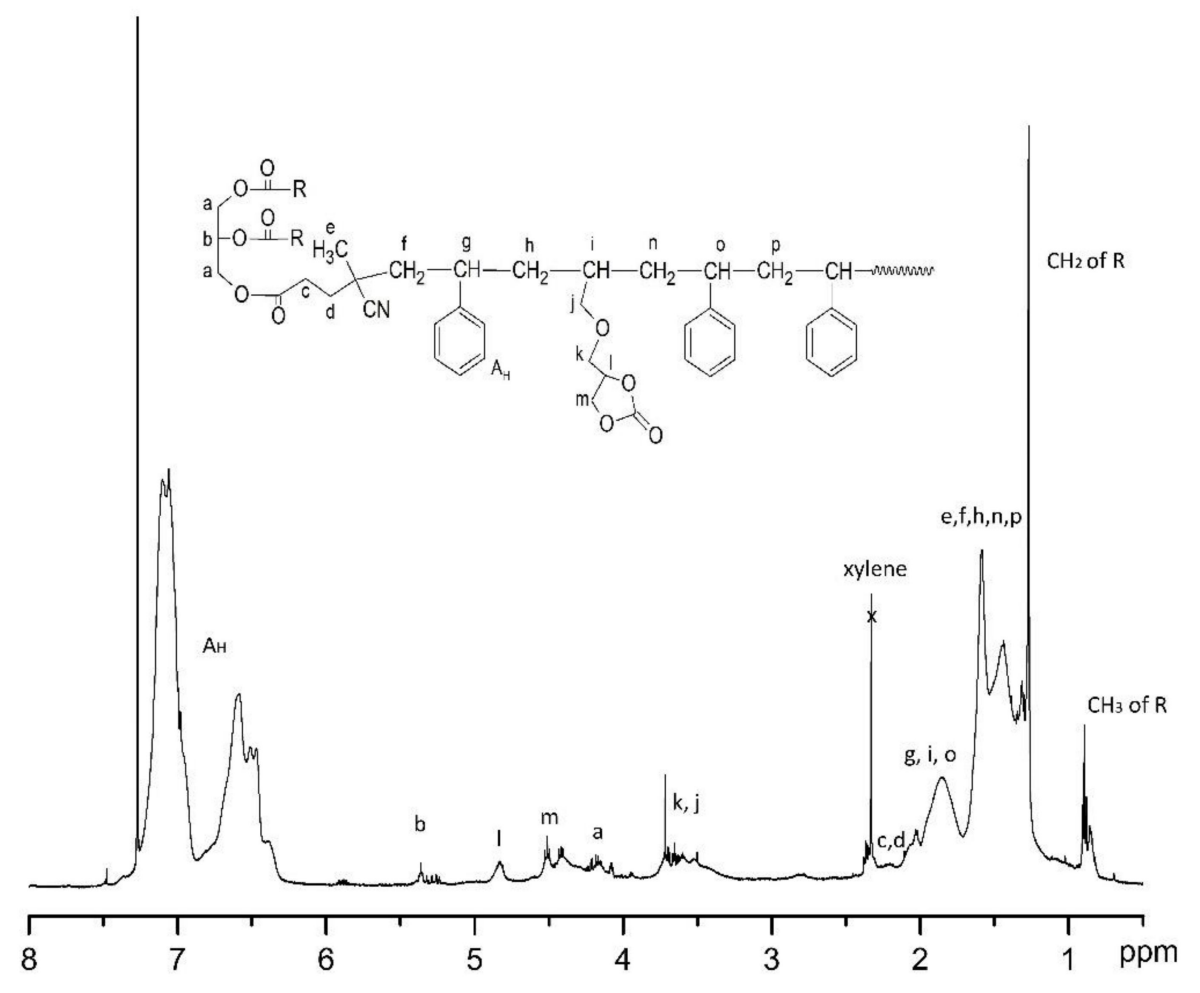
^1^H NMR spectrum of OBMI-St-AGC.

The ^1^H NMR spectrum of the OBMI-St-AGC is shown in Figure 6. The peaks at 7.4–6.2 ppm are attributed to aromatic protons of styrene, and the peaks at 5.36 and 4.3–4.1 ppm represent the protons of CH-O-CO-R and CH_2_-O-CO-R groups of oil moiety [9]. Additionally, the peaks at 4.82 and 4.55–4.35 ppm correspond to the protons of the cyclic carbonate groups of AGC [48,52,54]. The other peaks at 0.8–2.4 ppm represent the aliphatic -CH-, CH_2_-, and CH_3_ protons of the polymer [44,50,55]. The intensive peaks of vinyl groups of styrene and allyl groups of AGC appear in the range of 5–6.5 ppm [45,50]. Although the mol fraction of styrene/AGC was 60/40 in the feed, the peaks in this region almost disappeared. This shows that free monomers were removed efficiently by precipitation in methanol. In addition, these results showed that OBMI-St-AGC was successfully synthesized.

The copolymer composition was determined by the way as explained in the literature [48,56]. The integral area between 6.25 and 7.25 ppm, which indicates five adjacent hydrogen atoms on the aromatic ring of styrene, and the integral area between 4.9–4.75 ppm, which indicates CH proton of cyclic carbonate, were used to calculate the copolymer composition. The result of styrene/AGC was found as 93/7.

The molecular weight (Mn) and the polydispersity index (PDI) of OBMI-St-AGC were obtained by GPC, and they were found to be at 5576 g mol^−1^ and 2.7, respectively.

**Figure 7:**
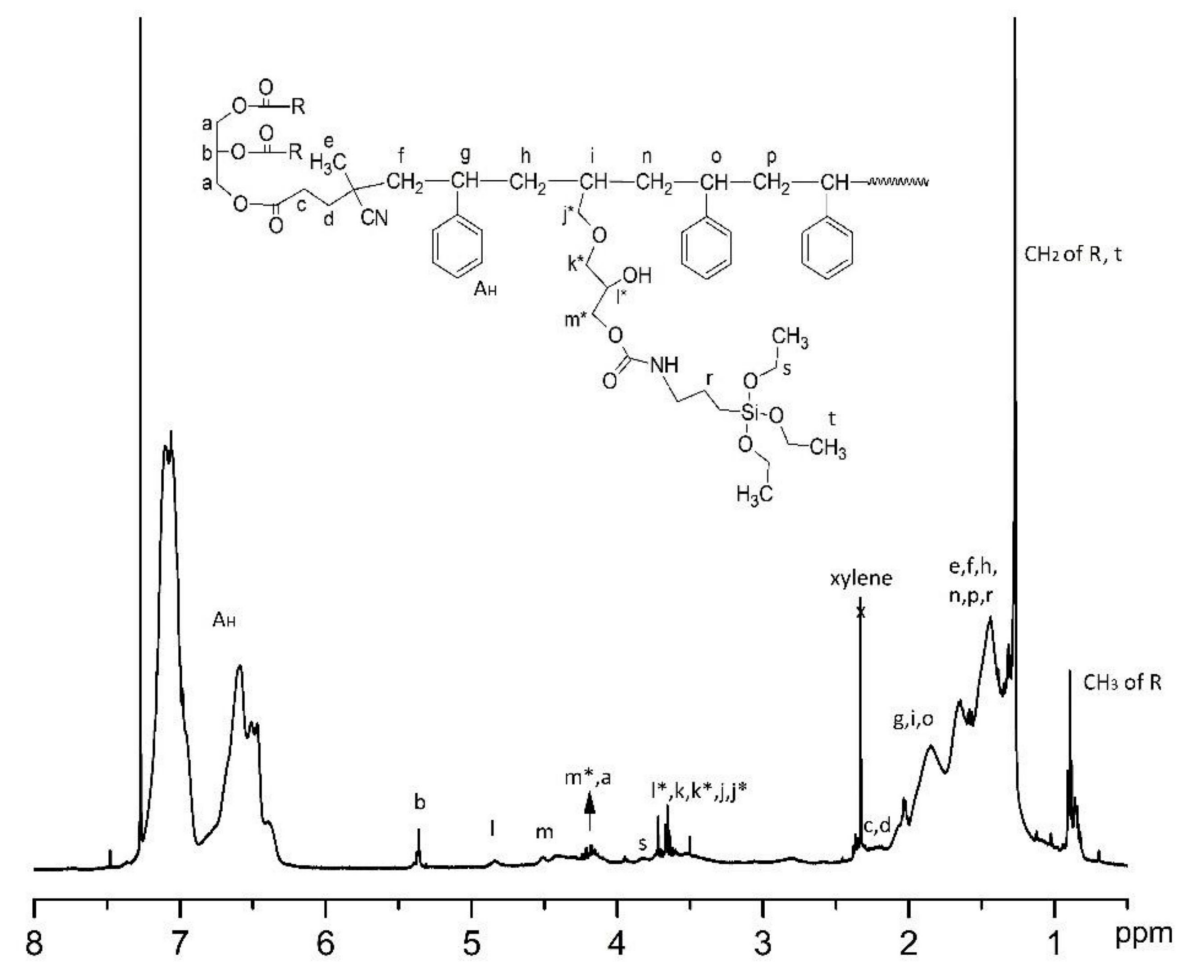
^1^H NMR spectrum of the OBMI-St-AGC-APTES.

After modification of OBMI-St-AGC with APTES, the polymer sample was also characterized by ^1^H NMR. As shown in Figure 7, the peaks’ intensity of the cyclic carbonate group became weaker at 4.82 and 4.55–4.35 ppm. The new peaks arose between 3.75 and 3.45 ppm, which represents the protons of CH_2_-O formed due to the ring opening of AGC [52,54,57]. These characteristic peaks of the polymer prove the successful achievement of APTES attachment to the OBMI-St-AGC structure. The percentage of the APTES content in OBMI-St-AGC-APTES was determined from the ^1^H NMR data by the same method as explained above [48,56]. It was found to be 0.6%.

**Scheme 2:**
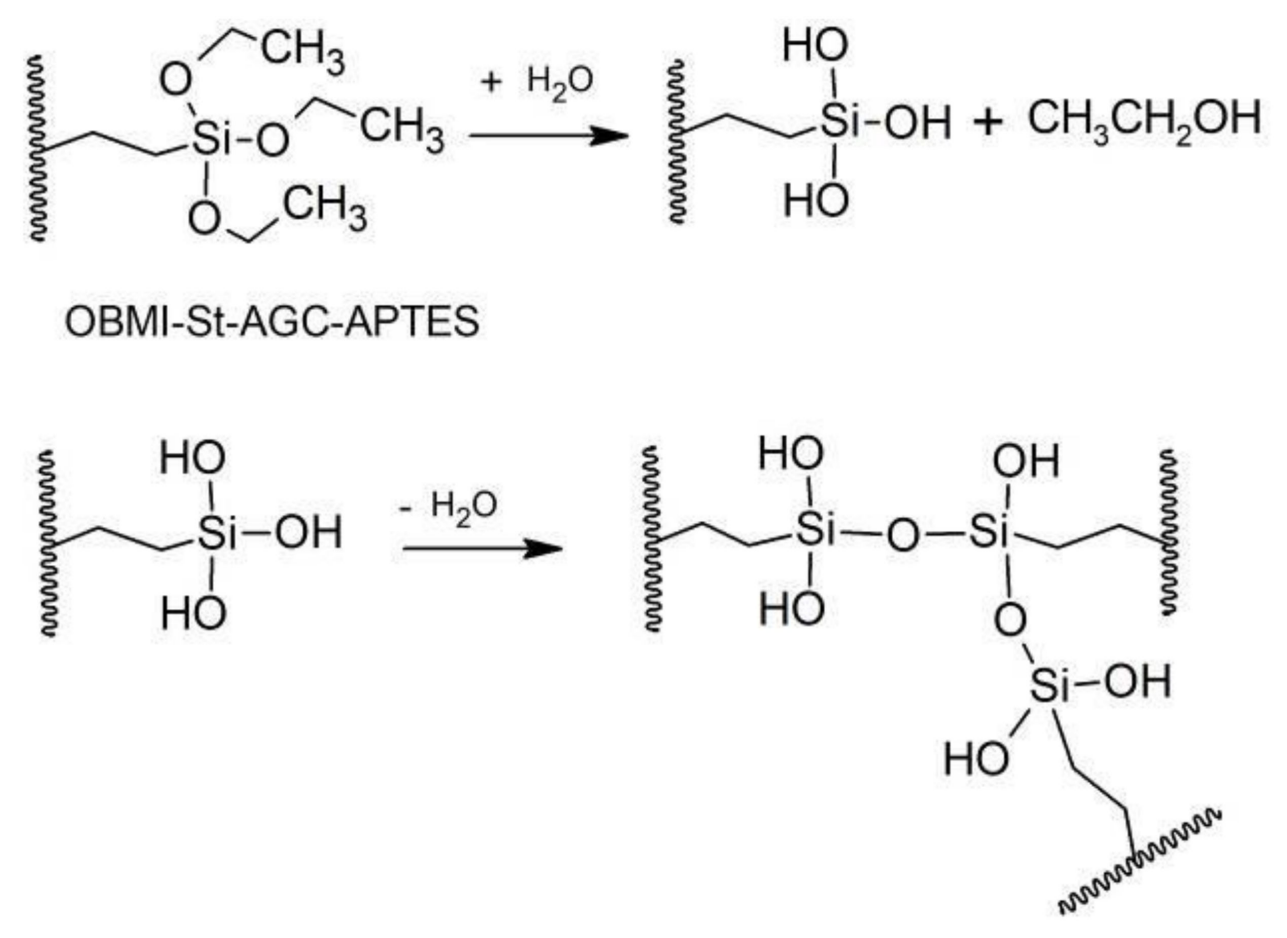
Representative sol–gel reaction.

The attachment of APTES was also proved by showing the crosslinking ability of OBMI-St-AGC-APTES through the sol–gel reaction. At the end of the sol–gel reaction, which was carried out under the explained condition in Section 2.7, the precipitated polymer sample in methanol was separated by the filtration, and the crosslinked structure was evaluated by FTIR analysis. The representative structure of C-OBMI-St-AGC-APTES by the sol–gel reaction is shown in Scheme 2.

**Figure 8:**
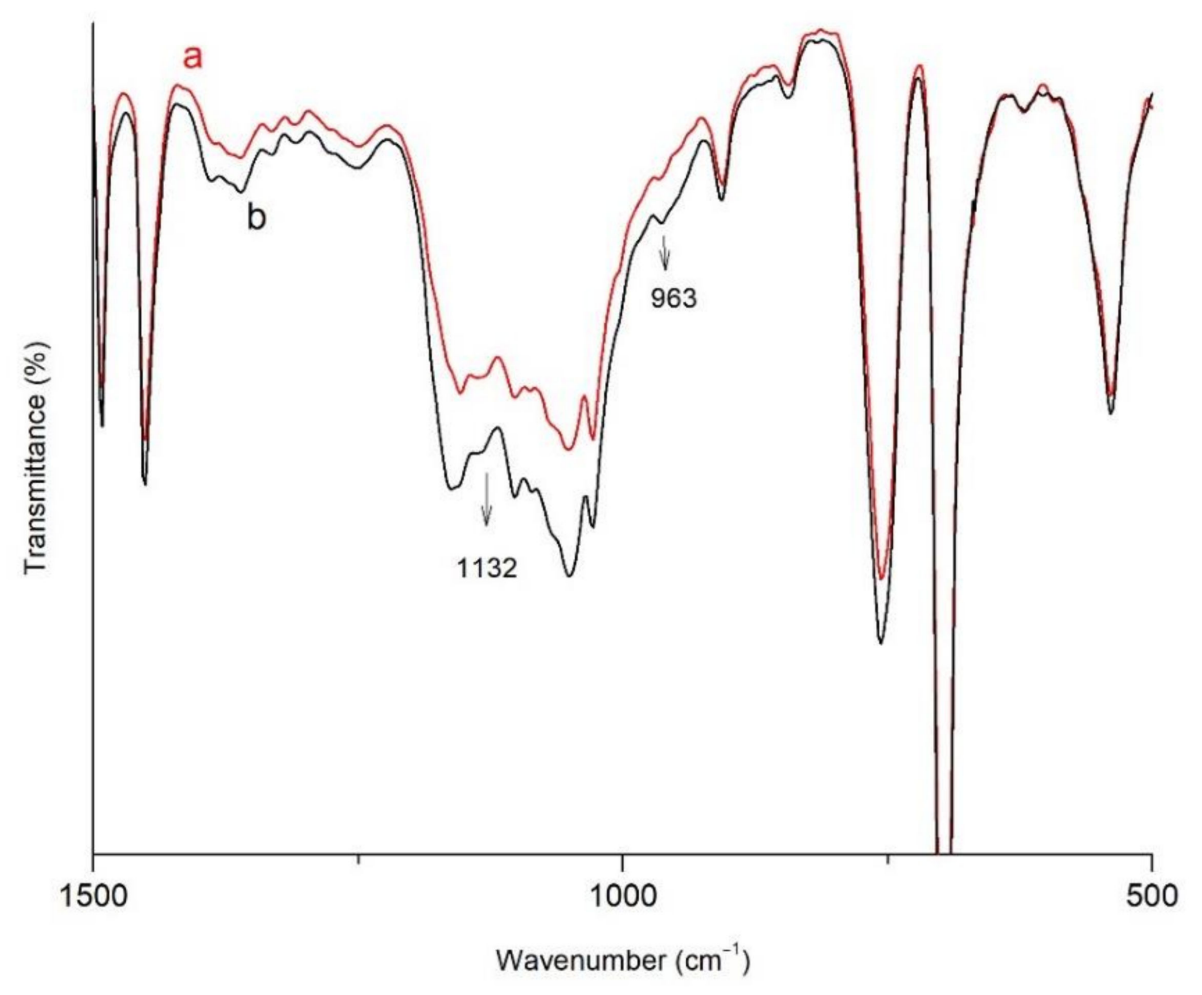
FTIR spectra of (**a**) C-OBMI-St-AGC-APTES and (**b**) OBMI-St-AGC-APTES.

The FTIR spectra of OBMI-St-AGC-APTES and C-OBMI-St-AGC-APTES samples are given in Figure 8. The peaks corresponding to the Si-O-Si, C-Si-O, and Si-O-C stretching are shown at the 1000–1200 cm^−1^ region in the spectra. The increase in peak intensity at 1132 cm^−1^ shows Si-O-Si bond formation through the sol–gel reaction [58,59]. Additionally, the intensity of the peak at 963 cm^−1^ (Si–O stretching vibration of Si–OCH_2_CH_3_) decreased to some extent after the sol–gel reaction, which represents the precipitated crosslinked structure [15]. Another indication of the crosslinked structure formation is the insolubility of the sample in xylene.

**Figure 9:**
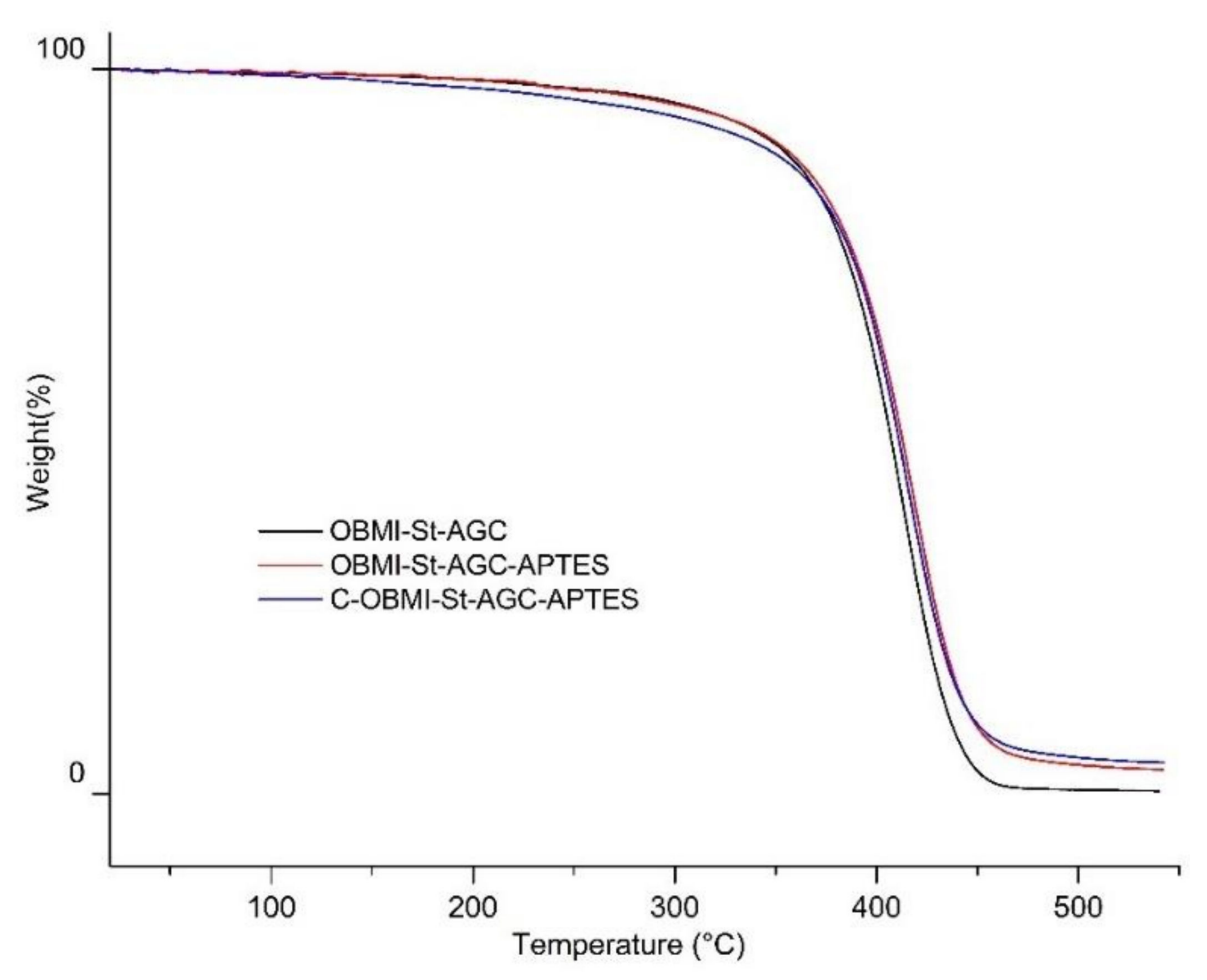
TGA thermograms of the polymer samples.

The TGA curves of the OBMI-St-AGC, OBMI-St-AGC-APTES, and C-OBMI-St-AGC-APTES samples are shown in Figure 9, and the weight loss percentage of the samples at 500 °C was found to be 99.48%, 96.05%, and 95.04%, respectively. Having less silane content, the C-OBMI-St-AGC-APTES sample exhibited slightly better thermal stability than that of OBMI-St-AGC. As shown in Figure 9, the char yield region started at 450 °C, and char yield values at 500 °C were 0.52%, 3.95%, and 4.96% for OBMI-St-AGC, OBMI-St-AGC-APTES, and C-OBMI-St-AGC-APTES, respectively. It is well known that the crosslinked structure of the polymer causes an increase in thermal resistance, as well as an increase in the char yield. Inorganic species present in the polymer sample remained in the char yield. The char yields of the samples were found in the order of C-OBMI-St-AGC-APTES > OBMI-St-AGC-APTES > OBMI-St-AGC. This is consistent with the above explanation since C-OBMI-St-AGC-APTES has a crosslinked structure and an inorganic moiety, while OBMI-St-AGC-APTES has an inorganic moiety but a crosslinked structure.

**Figure 10:**
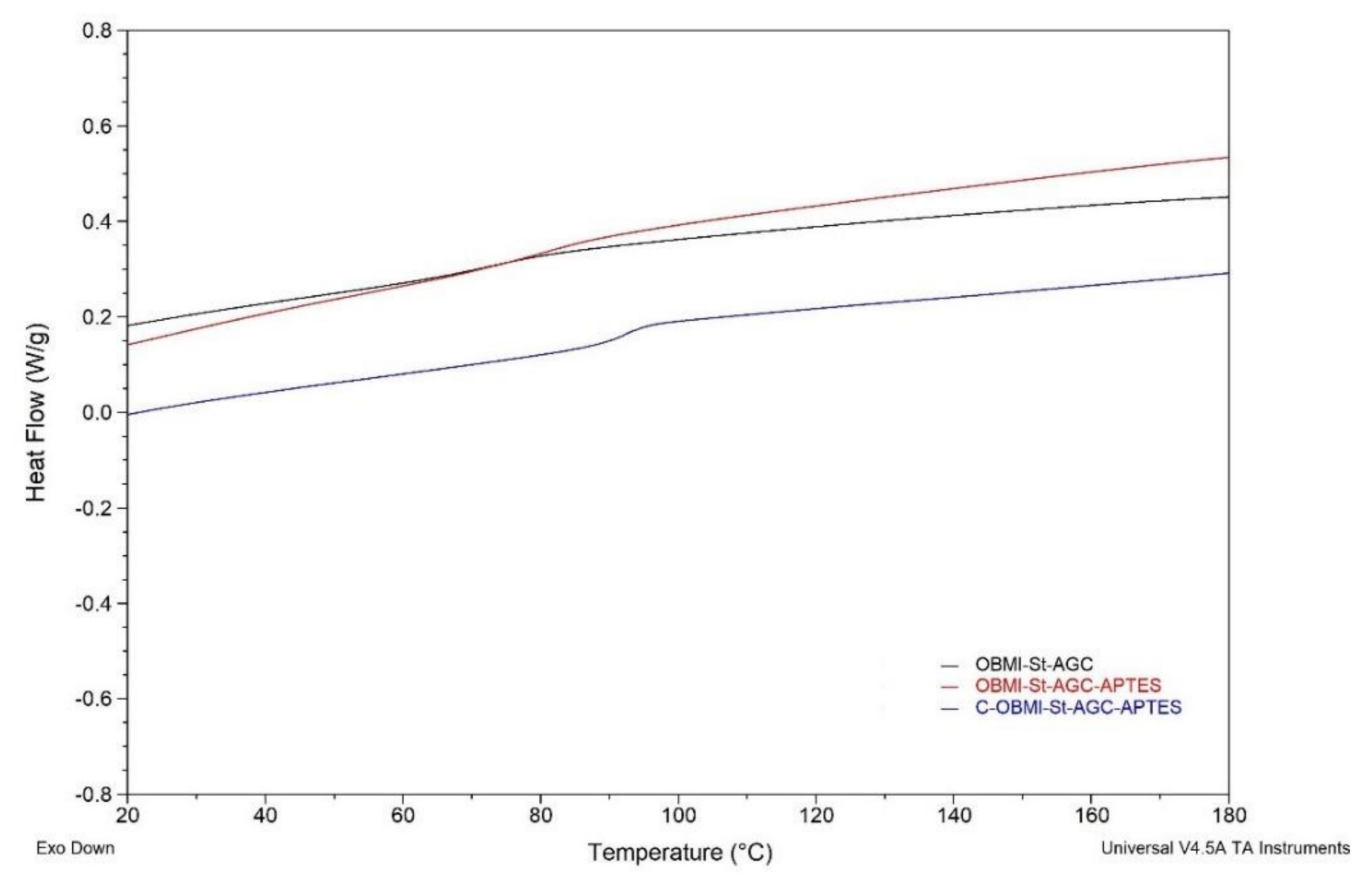
DSC thermograms of the polymer samples.

DSC thermograms of the OBMI-St-AGC, OBMI-St-AGC-APTES, and C-OBMI-St-AGC-APTES polymers are shown in Figure 10. The OBMI-St-AGC, OBMI-St-AGC-APTES, and C-OBMI-St-AGC-APTES samples exhibited a glass transition temperature (Tg) of 72.21 °C, 79.63 °C, and 92.4 °C, respectively. The C-OBMI-St-AGC-APTES sample showed remarkably higher Tg compared to that of the other samples. This is the expected result because the C-OBMI-St-AGC-APTES sample is obtained by the crosslinking of OBMI-St-AGC-APTES. In the literature, it was explained that the polymer samples showed higher Tg after silanization [60,61].

As explained above, initial film tests for the samples of OBMI-St-AGC and OBMI-St-AGC-APTES were applied. The films showed brittle characteristics. These results showed that OBMI-St-AGC could be used as a functional polymer, but for coating purposes, the soft segment should be increased in further studies.

## 4. Conclusions

In this study, a copolymer of styrene with AGC (St-co-AGC) modified with triglyceride oil was synthesized (OBMI-St-AGC). This polymer can be considered a functional polymer since it contains cyclic carbonate groups on its backbone. Moreover, OBMI-St-AGC was further reacted with APTES as an application of its functional property, and the alkoxysilane-containing polymer sample was successfully obtained. The characteristics of OBMI-St-AGC and C-OBMI-St-AGC-APTES were determined. OBMI-St-AGC could also be modified with other modifiers to incorporate the desired properties required by special applications. In this context, in further studies on coating materials, the soft segment should be increased in the structure in order to prevent the brittleness of the film.

## Data Availability

Not applicable.
